# Social factors associated with centenarian rate (CR) in 32 OECD countries

**DOI:** 10.1186/1472-698X-13-16

**Published:** 2013-03-08

**Authors:** Jong In Kim

**Affiliations:** 1 Division of Health and Welfare, Wonkwang University, Iksan, Republic of Korea

**Keywords:** Centenarian rate, Expenditure on health, Fixed-telephone, Human development index

## Abstract

**Background:**

Over the last fifty years, the number of centenarians has dramatically increased. The centenarian rate (CR) is representative of the general longevity prevalent in a nation; it indicates the number of individuals aged 100 years or above at a given date divided by the size of the corresponding cohort of a given age. Two important attributes of the CR (50–54) are that it reflects both unchanged age-specific fertility and the absence of migration in populations. It can generally be used in longevity-based evaluations of the broader concept of successful ageing. As such, this retrospective analysis of the social factors that contribute to the CR (50–54) may help to identify the factors associated with successful ageing.

This study estimates the CR (50–54) and elucidates the influence of social factors on successful ageing and the CR (50–54), examining 32 member countries of the Organization for Economic Co-operation and Development (OECD).

**Methods:**

The social indicators for this study were obtained from the United Nations database. The data for the analysis of centenarians in the 32 OECD countries were obtained from the world population prospects conducted by the United Nations. Associations between social factors and CR (50–54) were assessed using Pearson correlation coefficients and regression models.

**Results:**

Significant positive correlations were found between the CR (50–54) and the social factors of expenditure on health as a percentage of gross domestic product (HEGDP: r = 0.411, p < 0.021), general government expenditure on health as a percentage of total government expenditure (GGEH: r = 0.474, p < 0.006), the proportion of fixed-telephone subscriptions in the population (FTS: r = 0.489, p < 0.005), and the human development index (HDI: r = 0.486, p < 0.005). Finally, these CR (50–54) predictors were used to form a model of successful ageing, with higher HEGDP and GGEH as *health expenditure*, higher FTS as s*tandard of living*, and higher HDI as *social well-being* (R^2^ = 0.573, P < 0.025).

**Conclusions:**

The findings suggest that an increased CR (50–54) is affected by multiple social factors involved in successful ageing. Therefore, if they wish to improve their country’s CR (50–54), governments must strengthen their existing support services for the elderly through making improvements to standards of living, social well-being and through increased financing of the health sector.

## Background

In most countries, the number of centenarians represents the general longevity prevalent in the nation. The centenarian rate (CR) is defined as an estimate of the proportion of centenarians within a population [1]. It indicates the number of people aged 100 years or above at a given date divided by the size of the corresponding cohort of a given age. An advantage of using the CR (50–54) is that it enables the control of potential confounders that affect the number of centenarians, such as infant mortality, and overcomes the problem of migration inherent to changing nationalities [1]. Alternatively, the longevity index is calculated as the proportion between centenarians and the total population in a nation [2]. This value depends on population structure, and as such is affected by past fertility and migration; however, population changes are surprisingly slow to respond to dramatic life extension [3]. The CR (50–54), as an indicator of longevity, differs from the longevity index in that it is characterized by unchanged age-specific fertility and the absence of migration in its population.

Successful ageing has been the subject of many previous studies [4-9]; the longevity index was first suggested by Sachuk in 1970 [9]. The centenarian rate (CR) was suggested as an indicator of longevity in response to the imperfection of longevity index by Robine and Caselli [10], as a result of the longevity index having been used in certain countries [2,9,10]. Since an increasing number of centenarians may have been affected by social factors, using the CR (50–54) as a longevity index is a useful way to determine the social factors that are implicated in successful ageing in member states of the Organization for Economic Co-operation and Development (OECD). It is important that studies concerning longevity should focus on the social factors that may contribute to the CR (50–54) because successful aging is typically also associated with these factors. Therefore, this retrospective analysis of the social factors that contribute to the CR (50–54) may help identify the factors associated with successful aging.

In general, it is believed that the CR (50–54) is adequate to address the concept of successful aging. However, herein successful aging not only refers to people who have lived for 100 years or more but who also possess good functional capacity and general well-being [4,5]. Even though successful aging is a multidimensional construct with both objective and subjective dimensions, it invariably includes the absence of disease and disease-related disability along with an active engagement with life [6,7]. It involves an individual living to an advanced age with high physical function, preserved cognition, and continued social engagement [8]. These factors could also be commonly included in evaluations of longevity in successful aging research.

In the last fifty years, the number of centenarians has dramatically increased, particularly because of increasing life expectancy among the elderly [10]. However, along with longevity, successful aging is an important aspect of the health of centenarians. It is necessary for countries to promote successful aging to preserve elderly people’s health, their physical and mental abilities and their social well-being. This requires the possibility for the elderly to have appropriate use of the telephone to contact emergency medical facilities; government expenditure on health; and for the elderly to have strong human relationships, appropriate communication activities and a sense of satisfaction with their level of income and education. Finally, maintaining a healthy social role and a sense of purpose in old age are also major social factors involved in successful aging [10]. This paper therefore seeks to determine the association between such social factors and the number of centenarians in a population.

The primary aim of this study was to estimate the CR (50–54) as the prevalence of centenarians among the total corresponding cohort of a given age in the populations of 32 OECD countries. The secondary aim was to clarify the correlation between the CR (50–54) and the social factors involved in successful ageing by using a regression model.

## Methods

### Formulation of the CR (50–54)

This paper describes the conceptual study of the CR (50–54) within a framework of approaches obtained from the results of previous studies [1,10]. The study objective was to identify the differences in the social factors related to the CR (50–54) in 32 OECD countries. We utilized demographic databases that indicate the prevalence of centenarians from these countries to calculate the number of individuals aged 100 years or more at a given date divided by the size of a corresponding cohort of a given age. Thus, with regard to the last fifty years, the CR (50–54) in this study reflects the number of centenarians in the year 2011 divided by the number of people aged 50-54 years in 1961. Since this population is not likely to migrate or change nationalities, using this measure it is possible to control for other potential confounders such as infant mortality that affect the proportion of centenarians, which is particularly important when comparing rates between countries that have different infant mortality rates [1]. The CR (50–54) could be considered as showing the rate of living centenarians per 10,000 of the population.

### The framework of social factors

**Figure 1:**
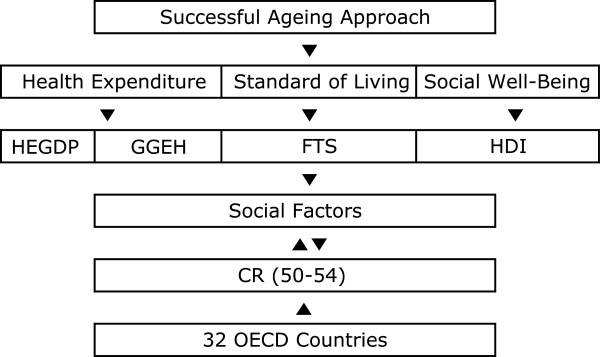
Description of theoretical framework.

This paper presents a conceptual study of social factors within a framework comprising the approaches and results of previous studies [11]. Successful aging in centenarians indicates the physical state of living for at least a hundred years with the absence of disease, alongside a mental state characterized by the preservation of functional capacity and social well-being, as exemplified through an active life and participation in society [4-8]. We believe that with respect to this, the social factors depicted in Figure 1 merit particular consideration within this framework.

Although the prevalence of chronic illness and disability increases with age, successful aging in the elderly population is widespread, and in OECD nations the elderly are generally in relatively good health. Indeed, the prevalence of disability among the elderly is declining, and expenditure on their care is increasingly concentrated at the end of life rather than during the preceding years of relative health [12].

There are multiple factors pertaining to *Health expenditure,* which contributes to the physical health aspect of successful aging, of which this study focuses on access to a high total expenditure on health as a percentage of gross domestic product (HEGDP), and general government expenditure on health as a percentage of total government expenditure (GGEH), While the impact of this factor has changed due to recent economic crises [13], a higher HEGDP and GGEH still entails greater access to health care and no reduction in hospital use among older people [14]. If there is a shortage of medical facilities and low expenditure on health, as would occur for instance during an epidemic, more hospital space becomes required at an additional cost; high mortality might result if hospitals cannot provide immediate treatment to patients. Increases in both the absolute number of elderly people and in their longevity will increase future health care expenditure [15].

The S*tandard of living* and *Human relationship* factors, which are contributors to the mental health aspect of successful aging and to high quality of life, were measured in the current study by the proportion of the population using fixed-telephone subscriptions (FTS). Although the relationship between cell phone usage and brain tumor incidence needs further epidemiological study [16], if an individual’s mental health improves or is maintained through telephone usage, this can be considered as having a beneficial effect on human relationships. In addition, a telephone subscription can be used for emergency calls in the case of accident. Furthermore, the very elderly are able to maintain social relationships with family and friends and receive more social support than do younger elderly adults [17]. FTS is also an indication of the developed telecom infrastructure networks that are apparent in developed countries, which also spend more on health and have a higher standard of living than is true for less developed nations [18]. Furthermore, the increased level of income that allows an individual to have such a subscription also suggests that they are able to pay for essentials such as food, shelter and healthcare.

Finally, *Social well-being* factors, which are related to how active an individual is in his or her society, were measured in this study using the human development index (HDI). This measures development by combining indicators of life expectancy, education, and income into a composite index [19] and shows a strong inverse relationship with the mortality ratio [20]. Furthermore, research has indicated a strong overall positive correlation between the level of human development and equality within this development [21], as for example assessed by the changing patterns of cancer according to varying levels of human development [19]. From this perspective, increases in future HDI values will increase both the absolute number of elderly persons and their longevity.

### Data and criteria

The data for the analysis of centenarians were obtained from the World Population Prospects, conducted by the United Nations [22]. The selection criteria for countries were based on the classification system applied by the OECD. We excluded Chile and Mexico from the CR (50–54) analyses because of insufficient information regarding the number of centenarians in these nations. A total of 32 countries were selected for this study. The social factors for this study were obtained from a dataset in the United Nations database [23]. The study used the following factors: (1) HEGDP, as a percentage of gross domestic products in 2006; (2) GGEH, general government expenditure on health, as a percentage of total government expenditure in 2006; (3) FTS per 100 inhabitants in 2011; and (4) HDI trends in 2011.

### Design of the model

In order to examine the association between the CR (50–54) in 32 OECD countries and the social factors involved in successful aging, we used a regression equation for each variable. A linear regression model was used to estimate the CR (50–54) in terms of the different factors. Thus, depending on the variables selected, the multivariate regression model yielded different results, which are discussed below. Of the three proposed models, models 1, and 2 assigned more significance to [HEGDP and GGEH], and [FTS and HDI] as being the best predictors of the CR (50–54). However, Model 3, which was based on Models 1 and 2, included [HEGDP and GGEH] and [FTS and HDI], and was perhaps the best model, as it considered access to the comprehensive model in the prediction variables of successful aging. Based on this model, we derived the following hypotheses: (1) Increases in HEGDP, GGEH, FTS, and HDI will lead to a corresponding increase in the CR (50–54) in the 32 OECD countries studied. (2) Decreases in HDI and GGEH will result in a corresponding decrease in the CR (50–54).

## Results

### Range of CR (50–54) and social factors

Table 1 presents the descriptive statistics for this range of CR (50–54) along with the social factors. The CR (50–54) of the 32 OECD countries ranged from 1.59 in Turkey to 116.78 in Japan. The mean HEGDP was 8.77%, with the lowest in Turkey (HEGDP 4.8%) and the highest in the United States of America (HEGDP 15.3%). The mean GGEH ranged from 9.9% in Poland to 19.3% in the United States of America, with a mean of 15.11%. The FTS ranged from 18.07 in Poland to 63.05 in Germany, with an overall mean of 42.91. HDI was the lowest in Turkey (HDI 0.7) and the highest in Norway (HDI 0.94), with a mean of 0.876 across all 32 OECD countries.

**Table 1:** Descriptive statistics of variable

**Variable**	**N**	**Mean**	**SD**	**Minimun**	**Maximum**
CR (50–54)	32	34.82	22.69	1.59	116.78
HEGDP	32	8.772	0.349	4.8	15.3
GGEH	32	15.11	2.839	9.9	19.3
FTS	32	42.91	12.59	18.07	63.05
HDI	32	0.876	0.046	0.7	0.94

SD: Standard Deviation.

### The CR (50–54) in the 32 OECD countries

Table 2 shows the CR (50–54) for all 32 OECD countries. The results indicate that among the 32 OECD countries, Japan had the highest CR (50–54), followed in order by Canada, Switzerland, Australia, France, the United States, Italy and then the United Kingdom. By contrast, the countries with the lowest CRs included Slovakia, the Czech Republic, and Turkey. Turkey had the lowest CR (50–54) of all the countries studied, with a value approximately 73 times lower than that of Japan.

**Table 2:** Centenarians rates in 32 OECD Countries

**OECD Countries**	**Centenarians** **2011 (A)**	**Aged 50–54** **1961 (B)**	**CR (50–54)** (A/B) * 10,000
Japan	49,457	4,235,088	116.78
Canada	603	860,932	70.04
Switzerland	218	345,347	63.12
Australia	3,367	56,535	59.56
France	17,337	2,925,417	59.26
United States of America	6,026	10,362,197	58.15
Israel	643	120,048	53.56
Italy	14,076	3,168,199	44.43
Iceland	34	7,963	42.69
Spain	6,449	1,661,656	38.81
New Zealand	48	126,651	37.89
Greece	1,737	466,364	37.25
United Kingdom	13,254	3,620,392	36.61
Denmark	931	294,494	31.61
Sweden	1,608	523,308	30.73
Netherlands	1,852	629,248	29.43
Norway	663	226,386	29.29
Estonia	197	75,769	26.01
Austria	13	501,994	25.89
Belgium	1,549	619,978	24.98
Ireland	39	156,295	24.95
Germany	12,682	5,365,255	23.64
Portugal	1,054	477,505	22.07
Finland	565	26,828	21.06
Slovenia	197	96,286	20.46
Republic of Korea	1,826	90,849	20.09
Luxembourg	38	23,513	16.16
Poland	2,683	1,676,958	15.99
Hungary	926	670,302	13.81
Slovakia	252	242,513	10.39
Czech Republic	547	6,908	7.92
Turkey	181	113,756	1.59

### Model of the prediction variables of the CR (50–54)

**Figure 2:**
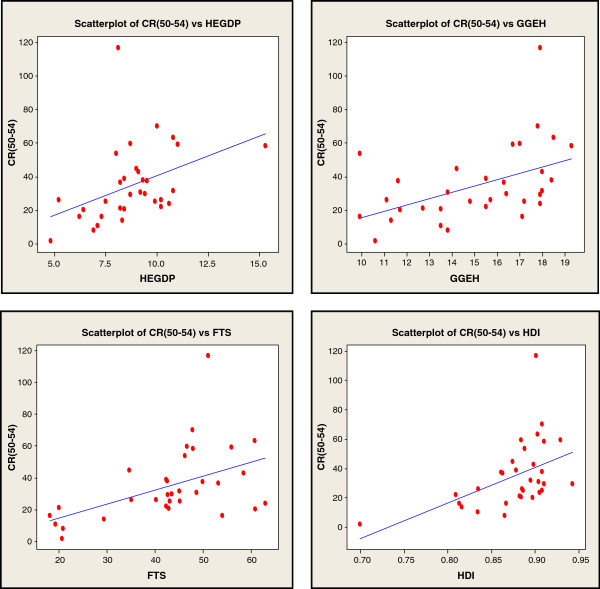
The scatterplots of the social factors for CR (50–54).

In order to investigate the direct relationships between the social variables involved in successful aging and longevity in the 32 OECD countries studied, we conducted a multiple regression analysis. Figure 2 and Table 3 show our analysis of the social factors related to the CR (50–54) in all 32 countries. Scatterplots of the country characteristics against the CR (50–54) are presented in Figure 2 in order to indicate the strength of the correlations between these social factors and the rate of successful aging. Significant positive correlations were found between the CR (50–54) and the entire social factors of HEGDP (r = 0.411, p < 0.021), GGEH (r = 0.474, p < 0.006), FTS (r = 0.489, p < 0.005), and HDI (r = 0.486, p < 0.005) (Figure 2). The regression analysis of the social factors found the strongest predictors among the three regression models (Table 3). Finally, the CR (50–54) predictors were used to form a model of successful ageing, including an higher HEGDP and GGEH as indicating *expenditure on health*, a higher FTS as indicating the s*tandard of living and human relationship quality*, and a higher HDI as indicating *social well-being* (R^2^ = 0.573, P < 0.025).

**Table 3:** Model of prediction variables of social factors for CR (50–54)

**Predictions variable**	**Coefficient**	**T-value**	**P-value**	**R**^**2**^
Model 1†				
HEGDP	2.078	0.859		
GGEH	2.862	1.702	0.017	0.494
Model 2‡				
FTS	0.561	1.635		
HDI	151.585	1.604	0.006	0.549
Model 3‡				
HEGDP	1.161	0.481		
GGEH	1.181	0.624		
FTS	0.436	1.181		
HDI	100.651	0.932	0.025	0.573

† Model 1: Expenditure on health.

‡ Model 2: Standard of living & Human relationship (+) Social well-being.

‡ Model 3: Expenditure on health (+) Standard of living & Human relationship (+) Social well-being.

## Discussion

In this study, we investigated the social factors that are associated with the CR (50–54). Based on the prevalence of centenarians, alongside other factors sourced from the national censuses of the 32 OECD countries considered in this study, we found that on an average, at present, there are 34.82 centenarians per 10,000 in these nations. The CR (50–54) was then used to formulate a model for identifying the social factors involved in successful aging.

The *Health expenditure* factor of HEGDP and GGEH, which reflects the physical health that is necessary for successful aging, was included in Models 1 and 3 and was found to be a significant factor in longevity. In the current study, the HEGDP in Turkey and GGEH in Poland were the lowest among all the countries, while that of the United States was the highest (Table 1). As such, the HEGDP is likely to be a major contributing factor to the low CR (50–54) in certain countries (Table 2). While the impact of this factor has changed following the recent economic crises [13], total gross domestic product continues to have a significant influence on life expectancy in OECD countries [24]. Between 1970 and 2005, the United States showed the largest increase in the percentage of gross domestic product devoted to health care among all of the OECD countries [25]. On the other hand, the poor and those living in economically less developed regions had the greatest risk of a high out of pocket burden for healthcare in Turkey [26]. Hence, health expenditure was associated with better outcomes in mortality [27]. The high mortality rate that stems from low expenditure on health and medical facilities is a well-known public health concern. Elderly populations who lack access to medical facilities may not have the same life expectancy as those centenarians who can avail themselves of medical facilities, irrespective of other factors. Hospital use can be explained on the basis of morbidity [28]. Furthermore, an ageing population combined with insufficient numbers of personnel available to provide medical care may potentially threaten the sustainability of a health care system [29]. However, the presence of appropriate medical facilities and adequate health expenditure is likely to help the elderly to maintain the requisite physical health for successful aging. Therefore, stronger positive correlations and CR (50–54) predictors were higher [HEGDP and GGEH], which may well indicate that HEGDP and GGEH is an important independent contributor to longevity (Figure 2; Table 3).

The S*tandard of living* and *Human relationship* factors of FTS, which can contribute to good mental health and to the high quality of life that is implicated in successful aging, were included in Models 2 and 3. Increases in FTS led to an increase in the CR (50–54), suggesting that this is a significant contributory factor to longevity and successful aging. Although cell phone usage needs further epidemiological evaluation [16], in the current study the FTS scores in Germany (63.05) and Switzerland (60.82) were the highest in any of the countries studied (Table 1), in agreement with their high CR (50–54) values (Table 2). Generally, OECD countries are likely to have high correlations between FTS and longevity because these variables are reflective of generally high levels of government investment, either in health or telecommunications infrastructure [18], as well as indicators of the high quality of life and high individual income of citizens in OECD countries. On the other hand, this means that if an individual’s mental health improves by using telephones, then human relationships which maintain social relationships with family and friends in the oldest-old [17] might have beneficial effects on mental health. Therefore, the study findings suggest that stronger positive correlations and predictors for higher FTS may be important independent contributors to longevity via the developed telecom infrastructure networks that are more common in developed countries that also spend more on health and have a higher standard of living than do less developed counterparts (Figure 2).

Finally, the s*ocial well-being* factor of HDI, indicating how involved the elderly are within their society, was included in models 2 and 3. An increase in HDI led to an increase in the CR (50–54), suggesting that the HDI is a significant contributor to longevity in successful aging. While Norway (0.943) and Australia (0.929) had the highest HDI among the countries studied (Table 1), which possibly contributes to the higher CR (50–54) in these nations, the HDI of Turkey was the lowest (Table 1), which is likely to be related to its having the lowest CR (50–54) (Table 2). Research indicates that HDI assessments are affected by the mortality, morbidity, and economic conditions of a country [20,30]. This suggests that a comfortable socioeconomic environment based on high levels of economic success and human development might help the elderly to maintain the requisite social health required for successful aging. Therefore, stronger positive correlations and predictors for higher HDI (Figure 2; Table 3) may indicate that the HDI value is an important independent contributor to longevity and increases in the absolute number of centenarians.

In the current study, the proposed three models that there is a little difference between the models (Table 3). It is caused by the fact that the four independent chosen variables are correlated (Figure 2). In the comprehensive analysis, however, if the governments for the proposed three models were to have a higher HEGDP and GGEH, a higher FTS, and a higher HDI, it would surely have an impact on the increase of CR (50–54) in all 32 OECD countries.

## Conclusion

After analyzing the CR (50–54) in 32 OECD countries, this study identified the following three important social factors as being implicated in successful aging: (1) a higher HEGDP, as a health expenditure factor related to good physical health (2) a higher proportion of the population using FTS, as a factor indicating a high general standard of living and higher social functioning in the elderly, and (3) a higher HDI value, as indicative of social well-being. From these results, it follows that governments must strengthen their existing support services for the elderly through achieving improvements to the standard of living and to social well-being and through increasing financing of the health sector, if they are to improve both the CR rate and the related prevalence of successful aging within the population.

## Competing interests

The author declares no competing interests.

## Authors’ contributions

JIK conceived of the study, data analysis, statistical analysis and drafted the manuscript. He made substantial contribution to analysis and interpretation of data. He contributed to the interpretation of results and the final writing of the paper. The author read and approved the final manuscript.

## Pre-publication history

The pre-publication history for this paper can be accessed here:

http://www.biomedcentral.com/1472-698X/13/16/prepub
