# Correction: Social factors associated with centenarian rate (CR) in 32 OECD countries

**DOI:** 10.1186/1472-698X-13-26

**Published:** 2013-08-13

**Authors:** Jong In Kim

**Affiliations:** 1 Division of Health and Welfare, Wonkwang University, Iksan, Republic of Korea

## 

After publication of the original article [1], the author noted that several of the figures in Table two (Table 1 here) were incorrect. The corrected table is reproduced here. We apologize for any confusion that this has caused.

**Table 1:** Centenarians Rates in 32 OECD Countries

**OECD Countries**	**Centenarians**	**Aged 50-54**	**CR (50–54)**
	**2011 (A)**	**1961 (B)**	(A/B) * 10,000
Japan	49,457	4,235,088	116.78
Canada	6,030	860,932	70.04
Switzerland	2,180	345,347	63.12
Australia	3,367	565,350	59.56
France	17,337	2,925,417	59.26
United States of America	60,260	10,362,197	58.15
Israel	643	120,048	53.56
Italy	14,076	3,168,199	44.43
Iceland	34	7,963	42.69
Spain	6,449	1,661,656	38.81
New Zealand	480	126,651	37.89
Greece	1,737	466,364	37.25
United Kingdom	13,254	3,620,392	36.61
Denmark	931	294,494	31.61
Sweden	1,608	523,308	30.73
Netherlands	1,852	629,248	29.43
Norway	663	226,386	29.29
Estonia	197	75,769	26.01
Austria	1,300	501,994	25.89
Belgium	1,549	619,978	24.98
Ireland	390	156,295	24.95
Germany	12,682	5,365,255	23.64
Portugal	1,054	477,505	22.07
Finland	565	268,280	21.06
Slovenia	197	96,286	20.46
Republic of Korea	1,826	908,490	20.09
Luxembourg	38	23,513	16.16
Poland	2,683	1,676,958	15.99
Hungary	926	670,302	13.81
Slovakia	252	242,513	10.39
Czech Republic	547	690,800	7.92
Turkey	181	1,137,560	1.59

## Pre-publication history

The pre-publication history for this paper can be accessed here:

http://www.biomedcentral.com/1472-698X/13/26/prepub
